# Influence of Impurities on the Electrochemical Upcycling of Biomass

**DOI:** 10.1002/cssc.202501887

**Published:** 2026-02-12

**Authors:** Lennart Sobota, Christoph J. Bondue, Kristina Tschulik

**Affiliations:** ^1^ Chair of Analytical Chemistry Faculty of Chemistry and Biochemistry Ruhr University Bochum Bochum Germany; ^2^ Max‐Planck‐Institut für Nachhaltige Materialien GmbH Düsseldorf Germany

**Keywords:** aldehyde oxidation, aldehydes, electrochemistry, gold electrode, HMF

## Abstract

The electrochemical oxidation of sugar‐derived compounds such as furfural and 5‐hydroxymethyl furfural (HMF) to the corresponding carboxylic acids is a crucial step in unlocking biomass as a renewable carbon feedstock. For instance, 2,5‐furandicarboxylic acid, the oxidation product of HMF, can replace crude‐oil derived terephthalic acid in the ubiquitous polymer polyethylene terephthalate. Hence, establishing an electrochemical process for the refinery of biomass requires oxidation of furfural and HMF with high current densities. It is therefore noteworthy that we show in this work that trace impurities undetectable by NMR spectroscopy and HPLC analysis impair the kinetics of electrochemical HMF and furfural oxidation. We therefore evaluate different methods for the removal of impurities from HMF and furfural that form during synthesis and storage of both chemicals. We find that purification by distillation of furfural and by recrystallization of HMF improves the kinetics of their electrochemical conversion best. Since both procedures can be adopted readily by other labs, the present work provides practical guidelines for the pretreatment of chemicals, which may also prove relevant for the development of processes at scale.

## Graphical abstract

5‐hydroxymethyl furfural (HMF) and furfural contain impurity levels that lie below the detection limit of common analysis techniques. Yet, these impurity levels have an adverse effect on the electrochemical conversion of these compounds. In this study, we show that distillation of furfural and recrystallization of HMF facilitates their electrochemical oxidation best.

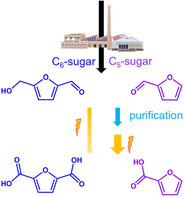

## 1 Introduction

2,5‐furan dicarboxylic acid (FDCA) is a promising building block for producing biomass‐derived polymers and it can substitute crude‐oil‐derived terephthalic acid in polyethylene terephthalate [1, 2, 3]. FDCA is obtained via the electrochemical oxidation of 5‐hydroxymethyl furfural (HMF), which in turn can be synthesized from biomass‐derived carbohydrates [4]. Currently, HMF is only commercially available with purities of approximately 99%, depending on the provider. The remaining 1% or less are impurities that form as side products during HMF synthesis [5] due to side reactions like rehydration or polymerization. Up until now, researchers in the field of HMF oxidation have rarely addressed the effect of these impurities and used HMF in their studies as received likely because these levels of impurities are not detected by ^1^H‐NMR spectroscopy [6, 7, 8, 9].

However, the negative impact of impurities on electrocatalytic reactions is well established: Hickling and Salt [10] and Bockris and Conway [11] observed a poisoning of platinum electrodes due to small amounts of arsenic and mercury during the hydrogen evolution reaction resulting in a significantly increased overpotential and, hence, reduces conversion rates. CO as an impurity that is produced by dehydrogenation in fuel cells after methanol cross‐over, blocks the activity of the cathode and reduces efficiency [12, 13]. Finally, in a previous work [14], we showed that the oxidation of aliphatic aldehydes is blocked at gold electrodes in the presence of strongly adsorbing anions like carboxylates.

With the present work, we highlight that impurities in commercially available HMF as well as furfural inhibit electrochemical oxidation at gold electrodes. Furthermore, we establish methods for the satisfactory purification of both substances.

## 2 Experimental

### 2.1 Substances

HMF (>99%), Furfural (99%), NaOH (99.99%), and NaClO_4_ (99.99%) were obtained from Sigma Aldrich. Sodium carbonate (99.5%) was purchased from Grüssing, the silica gel (0.06–0.2 mm, 60 A) from ACROS Organics and ethyl acetate (ACS. Reag. Ph. Eur.) and potassium permanganate (99%) from VWR Chemicals. Sand (low iron, general purpose grade), diethyl ether (>=99.5%), sulfuric acid (95%), and hydrogen peroxide (>30%) were obtained from Fisher Scientific.

Deionized water (0.055 µS/cm at 25°C) was obtained from a BarnstedTM GenPureTM xCAD Plus by Thermo Scientific.

Except HMF and furfural, all substances were used as received.

### 2.2 Purification of Aldehydes

Furfural was purified by vacuum distillation from sodium carbonate at 50 mbar and 125°C. After the first distillation, the furfural still featured a slight yellow color and, therefore, was distilled again under the same conditions, resulting in a colorless liquid.

HMF was purified by three different methods: distillation, chromatographic separation, and recrystallization. HMF was distilled in high vacuum at 200°C and was condensed in a Claisen condenser which was kept at 80°C and was finally captured in the receiving flask by cooling with liquid nitrogen. Chromatographic separation was performed over a silica bed of an approximate length of 30 cm and the bed was imbedded at the lower‐ and upper‐end with a layer of 2 cm sand. The eluent mixture consisted of 400 ml ethyl acetate, which was added to 2 l diethyl ether. The eluent from the captured HMF was removed using a rotary evaporator and dried under vacuum (0.09 mbar). For the recrystallization, 5 g of HMF were dissolved in approximately 75 ml of diethyl ether. The solution was first kept in a fridge at 5°C overnight to crystallize impurities. The supernatant was removed with a syringe and filtered through a PTFE‐syringe filter (0.22 µm, VWR) to remove suspended crystals of the impurities. The supernatant was then kept in a freezer at −27°C overnight to crystallize the HMF. The diethyl ether was then removed by decanting and the HMF was finally dried in a vacuum (0.09 mbar).

The purified furfural and HMF were stored under an argon atmosphere at −27°C. ^1^H‐NMR spectra of the unpurified and the purified substances were recorded in a solution of deuterated chloroform (99.8%, Deutero) at a Bruker DPX‐200 (200 MHz). High performance liquid chromatography (HPLC) analysis was performed with a P 6.1L pump, AS 6.1L Autosampler, a 100 µL sample loop, VU 4.1 column selection valve, CT 2.1 column oven, DAD 2.1L detector, and a Eurospher II 100‐5C18A 250 mm × 4 mm column equipped with a 100‐5C18A 5 mm ×4 mm column precolumn. The oven was kept at 25°C, the detector recorded at 275 nm and the eluant was changed linearly from 10% methanol in 5 mM ammonium formate solution to 30% methanol over 20 min with a constant flow rate of 0.5 ml/min.

### 2.3 Linear Sweep Voltammetry

The linear sweep voltammetry (LSV) was performed with a PGSTAT 128 (Autolab), a gold rotating disc electrode (RDE), (Ø = 8 mm, 99.99% purity and obtained along with a rotator from IPS Elektroniklabor), an Ag/AgCl (3.0 M KCl) reference electrode (SI Analytics), and a titanium rod (99.6%, Chempur) counter electrode. The electrochemical cell was divided into 3 compartments. The reference electrode was separated by a Luggin capillary with an additional glass tap filled with 1 M NaClO_4_ to prevent chloride leaking into the electrolyte and the counter electrode was isolated by a glass frit. The aqueous solution for the LSV consisted of 10 mM NaOH and 100 mM NaClO_4_ to which the corresponding amount of reactant was added. Prior to recording the LSV, electrochemical impedance spectroscopy was performed to determine the solution resistance and to correct the ohmic drop (80%) via positive feedback loop. The RDE was rotated at 60 rpm to limit the overall current and thus the iR drop. A scan rate of 10 mV/s was used.

The electrochemical cell was cleaned by immersing it in 5 mM KMnO_4_ in 0.5 M H_2_SO_4_ for at least 10 h. Directly before use, the glassware was then rinsed with deionized water. Remaining MnO_2_ was removed by rinsing with diluted acidic hydrogen peroxide and subsequent rinsing with ultrapure water (Thermo Scientific Barnstead GenPure xCAD Plus, conductivity: 0.055 µS).

## 3 Results

### 3.1 5‐Hydroxymethyl Furfural

**FIGURE 1:**
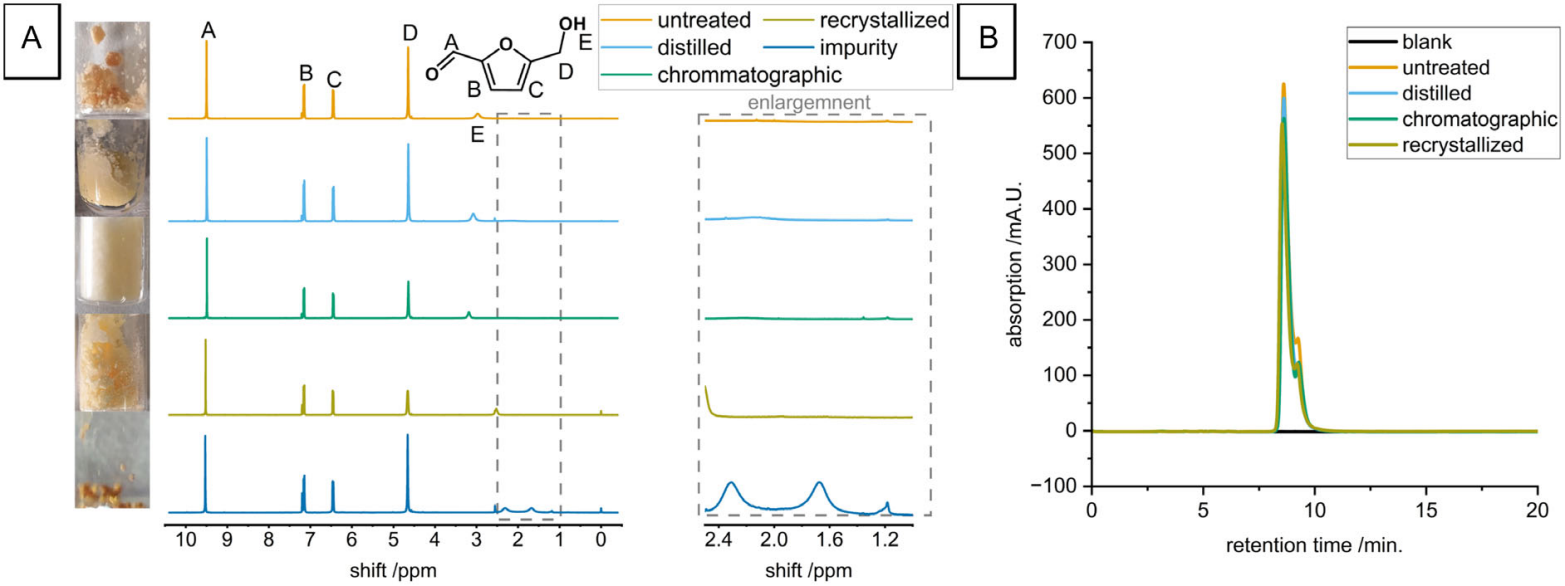
(A) Photos and ^1^H‐NMR spectra of different HMF samples with peak assignment. From top to bottom: untreated, distilled, chromatographically purified, recrystallized HMF, and residue obtained while dissolving HMF in diethyl ether. (B) HPLC chromatograms of the respective HMF samples.

Figure 1A shows the ^1^H‐NMR spectra of untreated and purified HMF. Relative signal intensities and signal positions are in good agreement with previously reported spectra for all samples [15]. None of the spectra shows a signal that indicates the presence of impurities. The only exception is the distilled sample, which features an additional signal with a shift of 2.61 ppm, indicating the degradation of HMF during distillation. This finding is supported by the HPLC analysis (Figure 1B), where no additional signals are observed. Nevertheless, the as‐received HMF cannot be considered pure: the bottom of Figure 1A shows a ^1^H‐NMR spectrum of the substance that remained as a dark brown residue while dissolving the HMF in diethyl ether. This undissolved substance indicates the presence of impurities in as‐received HMF and the unsteady baseline in the 1.5–2.5 ppm range shows that these impurities feature a structure strongly deviating from that of HMF. Accordingly, the untreated HMF must contain side products of synthesis (e.g., alkenes, ethers, or humic acids [5, 16, 17]) in a concentration that is not detected by standard analytical techniques, such as ^1^H‐NMR spectroscopy or HPLC analysis.

**FIGURE 2:**
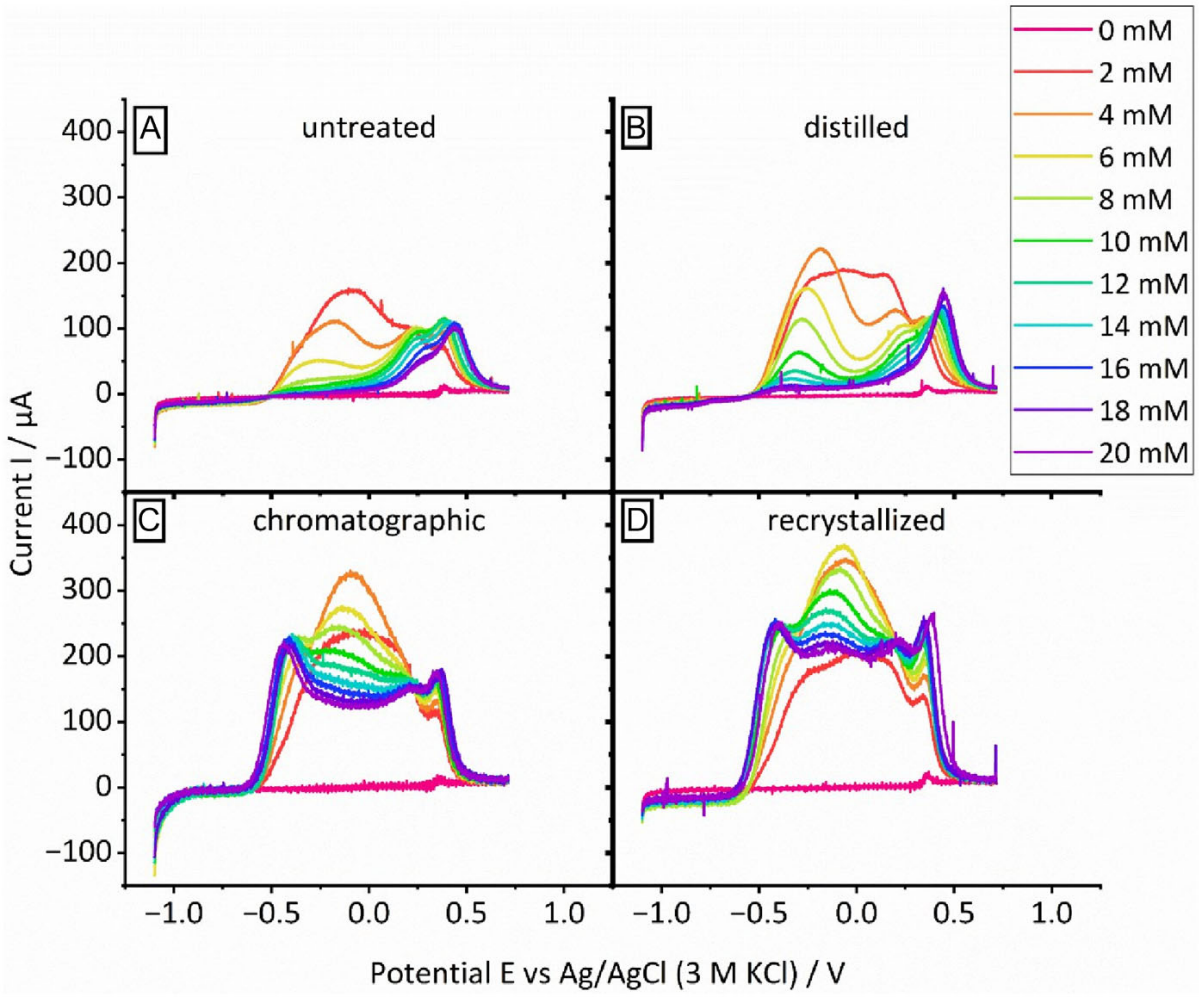
LSV of (A) untreated, (B) distilled, (C) chromatographically purified and (D) recrystallized HMF; electrolyte: 10 mM NaOH and 100 mM NaClO_4_, scan rate: 10 mV/s, Au RDE at 60 rpm rotation speed, HMF concentration was increased successively, as stated in the legend.

The results displayed in Figure 1 highlight that techniques such as NMR and HPLC, which are commonly used in the analysis of organic/biomass‐derived compounds [18], feature insufficient sensitivity to assess the purity of biomass‐derived compounds. However, as a surface‐sensitive technique, heterogeneous electrocatalysis can be sensitive to impurity levels significantly below the detection limit of either of those techniques. This is clear from Figure 2, where the purification method exerts a clear effect on the current‐potential curves obtained in the presence of HMF. That is, Figure 2 compares how the LSV (anodic sweep) at a gold electrode evolves when the HMF concentration is gradually increased. For all HMF samples (untreated, purified by different means), an oxidation process is observed in the potential region between −0.5 and 0.5 V. In a previous paper, we argued that in this potential range, the oxidation of the aldehyde functional group of HMF takes place [14, 19], whereas the oxidation of the alcohol functionality is limited to the potential range positive of 0.4 V [14].

It is noteworthy that the current drops to almost zero in the potential range between −0.5 and 0 V when the concentration of untreated or distilled HMF is increased (Figure 2A,B). This indicates a negative reaction order in HMF, apparently. However, this behavior is not observed or strongly decreased when HMF is purified by chromatography or recrystallization (as seen in Figure 2C,D). Hence, the results of Figure 2 show that impurities which accumulate in the electrolyte when the HMF concentration is increased suppress the oxidation of the aldehyde functional group, whereas the oxidation of the alcohol functional group is not affected. Conversely, the results of Figure 2 also suggest that purification of HMF by chromatography and recrystallization removes impurities more effectively than distillation, which is in line with the NMR spectra shown in Figure 1A. Accordingly, the poisoning effect observed in Figure 2A,B is not present or is less pronounced, as the overall content with impurities is reduced. We assign the effect of the cleaning method on the shape of the LSV to the effectiveness with which this method removes impurities from the reactant.

### 3.2 Furfural

**FIGURE 3:**
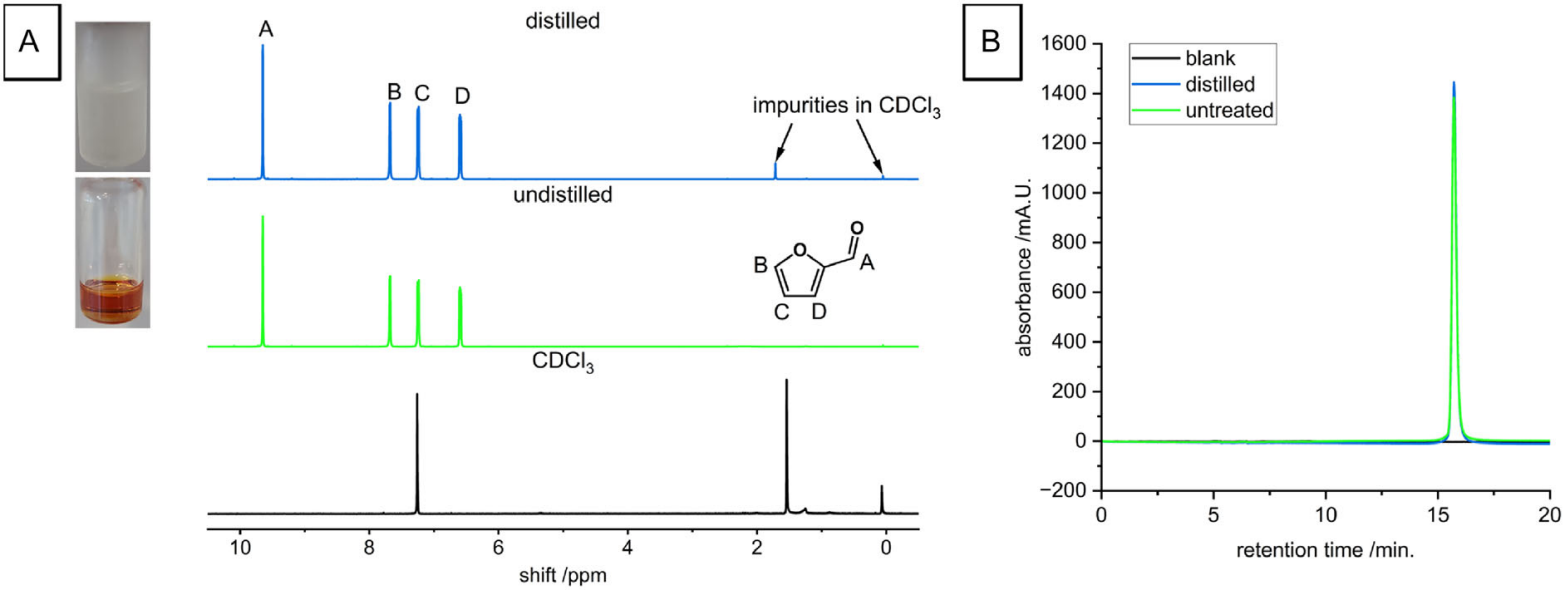
(A) Photos and ^1^H‐NMR spectra with peak assignment of untreated and distilled furfural. (B) HPLC chromatograms of distilled and untreated furfural.

Similar effects of purification are observed for the electrochemical oxidation of furfural, which features a strong structural similarity to HMF. The as‐received furfural is of brownish–yellowish color and turns colorless after distillation. This shows that the as‐received furfural contains impurities that are removed upon distillation (c.f. images in Figure 3A). Yet, as for the previous example, the ^1^H‐NMR spectra of distilled and undistilled (untreated) furfural show identical signals (Figure 3A). Both spectra only feature four signals, which correspond to the four hydrogen atoms of furfural [16]. Note that the additional signals in the distilled furfural originate from deuterated chloroform. Similarly to the HPLC analysis of HMF, the HPLC analysis of as‐received and distilled furfural also resulted in a chromatogram that only featured one signal caused by the furfural. This means that, also in this case, the contained impurity is not detected by one of the standard analytical techniques used.

**FIGURE 4:**
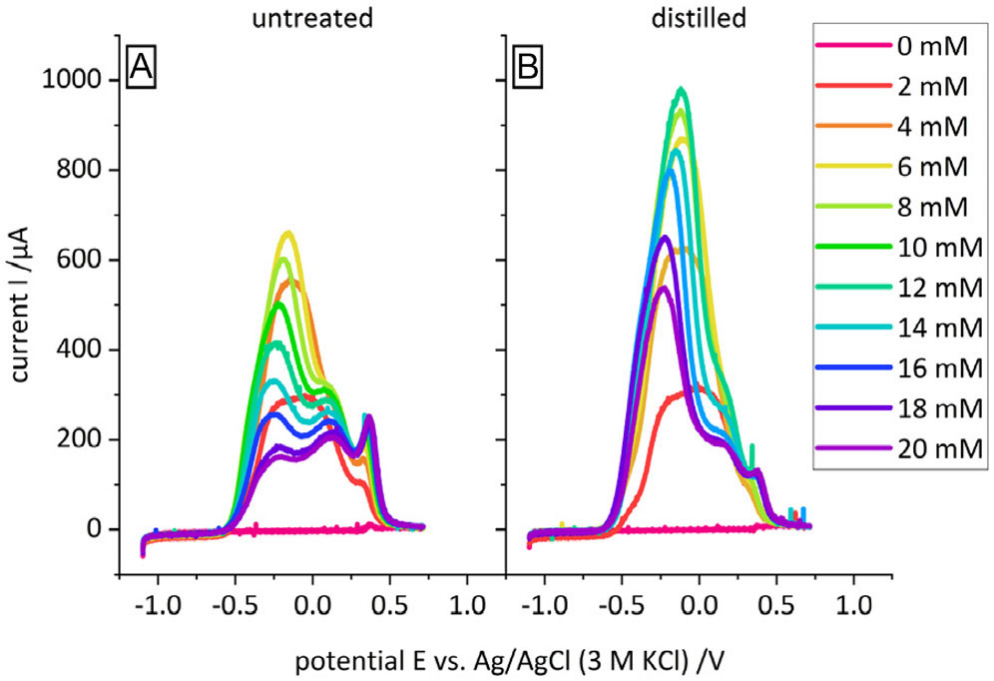
LSV of (A) untreated and (B) distilled furfural. LSVs were recorded at 10 mV/s at a Au RDE at 60 rpm in 10 mM NaOH and 100 mM NaClO_4_, the furfural concentration was increased successively as indicated in the legend.

In Figure 4, we compare the LSV obtained in the presence of undistilled and distilled furfural, respectively. When the electrolyte features a concentration of 2 mM furfural, the LSV for the unpurified and purified state does not differ: soon after passing the onset potential of furfural oxidation at −0.5 V, the current enters a plateau of 300 mA, which corresponds to the diffusion‐limited current of 325 mA as determined from the Levich Equation [20, 21]. At a potential of 0.5 V, the oxidation of furfural also stops due to the formation of catalytically inactive gold oxide on the electrode [14, 19]. By increasing the furfural concentration to 4 mM, the obtained plateau current doubles for both samples.

However, upon further increase of furfural concentration, differences appear. The current response for both cases changes and transforms into a peak‐shaped response with up to three peaks. The origin of this alternated current response is not clear yet. More importantly, the oxidation current observed for the distilled and undistilled furfural sample starts to differ. Depending on the concentration, an oxidation current for the distilled (Figure 4B) samples is observed that is between 1.3 and 2.4 times larger than undistilled furfural (Figure 4A). Furthermore, the highest obtained current for undistilled furfural already drops after reaching a concentration of 6 mM and for distilled furfural, the currents are still increasing until 12 mM. The first of the three peaks remains visible for the distilled furfural and disappears for the undistilled furfural at concentrations higher than 14 mM. Hence, we demonstrated that also for as‐received furfural of highest commercially available purity, the removal of impurities facilitates the electrochemical oxidation of this compound, which falls in line with our results for HMF oxidation.

## 4 Discussion

**SCHEME 1:**
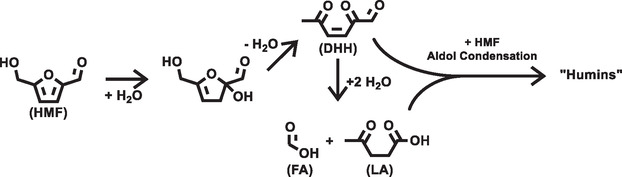
Mechanism of humin formation from HMF. In the first step HMF is hydrated, which can be catalyzed by protons or hydroxide. The hydrated HMF can form DHH, which can react further to LA. Finally, HMF, LA, and/or DHH can undergo aldol condensation to form humins, that is structurally ill‐defined polymers.

The bottom of Figure 1A shows the ^1^H‐NMR spectrum of the residues left after dissolving HMF in diethyl‐ether. Although the presence of broad signals at 2.3 and 1.7 ppm indicates the accumulation of impurities, they provide little insight into the nature of these impurities. Attempts to remove HMF, which still dominates the ^1^H‐NMR spectrum, were not successful. A clean spectrum that would hold more structural information of the impurities in HMF or in furfural was therefore not obtained. However, it is well established that humins and levulinic acid form as byproducts during the synthesis of HMF via the acid catalyzed dehydration of sugars [22, 23, 24, 25]. As shown in Scheme 1, addition of water and the ensuing opening of the furan ring leads to the formation of 2,5‐dioxo‐6‐hydroxyhexanal (DHH), which reacts further to levulinic acid (LA), and formic acid. Also hexoses can form DHH and LA [12, 24, 25], which engage together with HMF in aldol condensation leading to structurally ill‐defined polymers that are commonly referred to as humins. A similar mechanism as shown in Scheme 1 exists for the degradation of furfural to humins [12]. It stands therefore to reason that DHH, LA and/or humins are the relevant impurities found in HMF and furfural.

Our results presented in Figures 2 and 4 highlight that removal of impurities from HMF and furfural has a positive effect on the electrochemical oxidation of the aldehyde functional group of both compounds. However, the effect of reactant purification also depends on the reactant concentration in the electrolyte: Distillation of furfural has essentially no effect on the reaction kinetics when the furfural concentration is as low as 2 mmol. This changes when the furfural concentration increases to 10 mmol. In this case distillation improves the achievable current density by a factor of 2. We assign this to the impurity concentration in the electrolyte that increases with the reactant concentration. The exact mechanism by which impurities impair the oxidation of HMF or furfural is not clear, but it stands to reason that polymers such as humins adsorb on the electrode surface and render active surface sites inaccessible for the reactant. Other potential impurities such as DHH and LA may undergo electrolyte‐induced aldol condensation and may deactivate the electrode after precipitation as well.

Although purification facilitates the oxidation of furfural and HMF in our experiments that occur on a short time scale, it is not expected that these results are transferable to long‐term electrolysis experiments in which the organic reactant and hydroxide reside in the same phase. That is, the degradation mechanism shown in Scheme 1 can be catalyzed both by protons and hydroxide [26]. Initial removal of impurities is not expected to improve current density in long‐term electrolysis experiments, since the same impurities should form due to electrolyte‐induced degradation processes. Our results are nonetheless relevant for those electrolysis approaches in which degradation processes are suppressed effectively. This can for instance be achieved by separating organic reactant and hydroxide into immiscible phases [27]. Electrolysis is then conducted at a porous electrode placed at the formed liquid|liquid phase boundary. In such a setup reagent purification has a lasting effect on the achievable current density.

## 5 Conclusion

Our work shows that impurity levels undetectable by NMR spectroscopy or HPLC analysis suppress the electrochemical oxidation of biomass‐derived compounds such as HMF and furfural. Purification of both compounds facilitates therefore their electrochemical conversion. Among the tested purification methods, recrystallization enhances the electrochemical oxidation of HMF the most. Purification of furfural via distillation has a strong effect on the electrochemical oxidation of the aldehyde functionality as well. Since both purification techniques are often used in large‐scale applications [28, 29], our results hold relevance for the development of electrochemical processes of biomass upgrading where purification will be essential to maximize current densities and help to reach industrial relevant currents.

## Funding

Deutsche Forschungsgemeinschaft (EXC 2033‐390677874‐RESOLV; 388390466 – TRR 247; 54035022).

## Conflicts of Interest

The authors declare no conflicts of interest.
